# Preliminary experience with laparoscopic common bile duct exploration

**DOI:** 10.1186/s12893-017-0225-y

**Published:** 2017-03-31

**Authors:** Asaad F. Salama, Mohamed E. Abd Ellatif, Hesham Abd Elaziz, Alaa Magdy, Hisham Rizk, Magdy Basheer, Wisam Jamal, Ibrahim Dawoud, Ayman El Nakeeb

**Affiliations:** 1 Department of Surgery, Theodore Bilharz Research Institute, Cairo, Egypt; 2 Department of Surgery, Al-Jahra Hospital, Jahra, Kuwait; 3 Department of Surgery, Mansoura University Hospital, Gihan El Sadat St., Mansoura, 35511 Dakahlia Egypt; 4 Department of Surgery, Hafr Elbatin Central Hospital, Hafr Elbatin, Kingdom of Saudi Arabia; 5 Gastroenterology Surgical Center, Mansoura University, Mansoura, Egypt; 6 Department of Surgery, University of Jeddah, Jeddah, Kingdom of Saudi Arabia

**Keywords:** Laparoscopic, Choledocholithiasis, Cholelithiasis

## Abstract

**Background:**

Herein we present our experience with laparoscopic common bile duct exploration (LCBDE) in managing common bile duct stones.

**Methods:**

Data of 129 consecutive patients who underwent laparoscopic cholecystectomy (LC) and LCBDE done at our institutes from April 2011 through June 2016 were prospectively recorded and retrospectively reviewed.

**Results:**

Since 2011, 3012 laparoscopic cholecystectomy were performed at our institutes, intraoperative cholangiogram (IOC) was done in 295 (9.8%) patients which detected choledocholithiasis in 129 (4.3%) of them. LCBDE was successful to clear the common bile duct (CBD) in 123/129 (95.4%). Six patients underwent postoperative endoscopic retrograde cholangiopancreatography (ERCP) because of incomplete CBD clearance (4 cases), symptomatic stenosed papilla (2 cases). LCBDE was performed in 103 patients via trans-cystic approach and choledochotomy one in 26 patients. In the choledochotomy group, seven patients had primary closure of the CBD, CBD was closed over T-tube in nine patients whereas the remaining 10 patients the CBD was closed over antegrade inserted stent. The median time of hospital stay was 4 (range; 1–15) days. No patients showed retained CBD stones with mean follow up was 9 ± 3.4 months.

**Conclusion:**

LCBDE is a safe and cost effective option for CBD stones in short-term outcome and can be performed provided proper laparoscopic expertise and facilities are available.

## Background

Approximately 9–16% of patients who have gall bladder stone harbor common bile duct stones [1, 2]. Open cholecystectomy with CBD exploration and clearance was the standard treatment of CBD stone associated with gall bladder stone [3, 4]. With increasing experience in laparoscopy and as laparoscopic cholecystectomy (LC) became the standard treatment for gall bladder stone, management of CBD stones strategy has been changed and endoscopic retrograde cholangiopancreatography (ERCP) got the upper hand in this field preceded with or followed by LC (two stage procedure) [5]. It has been reported that endoscopic sphinctrotomy has up to 15% morbidity rates and 1% mortality rates and it also increase the cost cause of two stage procedure and it may cause recurrent ductal stones and stenosis of the papilla with cholangitis. Accordingly and in addition to the surge in laparoscopic experience, LCBDE becomes a potential option for managing CBD simultaneously with LC (one stage procedure) [6].

We aimed in this retrospective study to evaluate our initial experience in using LC and LCBDE as a single stage procedure for management of CBD stones with gall bladder in situ.

## Methods

From April 2011 to June 2016, we performed 3012 laparoscopic cholecystectomy at our institutes (Theodore Bilharz Research Institute, Egypt, Mansura University Hospital-Egypt, Hafer Albatin Central Hospital, KSA, University of Jeddah-KSA, Al-Jahra hospital, Kuwait). Two hundred ninety-five patients had the indications to undergo intraoperative cholangiogram which documented presence of CBD stones in 129 patients. These 129 patients’ data were prospectively collected and enrolled in this study.

### Patients’ selection

Patients with history of classic biliary pain and jaundice had underwent routine preoperative preparation including complete blood count (CBC), coagulation profile, kidney functions, liver function tests (LFTs), abdominal ultrasonography and routine pre-anaesthetic evaluation to determine ASA status. Magnetic resonance cholangiopancreatectomy (MRCP) was done in 31 patients. Intraoperative cholangiogram (IOC) was performed for patients with gall bladder stones who had one or more of the following; classic biliary pain, history of biliary pancreatitis, unexplained LFTs abnormalities, and radiologic evidence of dilated biliary tree or CBD stones. LCBDE was done if there were abnormal intraoperative cholangiogram findings (one or more) such as filling defect, wide diameter biliary tree or abnormal passage of contrast into the duodenum. This study was approved by institutional research board (IRB). An informed consent was obtained from all patients after been provided with information about their disease, the current updated treatment options and possibility of conversion to conventional one, T-tube insertion and postoperative ERCP if indicated.

On June 2016, the 129 patients’ data sheets were retrospectively reviewed in respect to their preoperative, operative notes and postoperative data. Preoperative data include; age, gender, body mass index (BMI), ASA status, liver function tests (LFTs), ultrasonography and magnetic resonance cholangiopancreatectomy (MRCP) if done. Operative data include; operative time, transcystic or transcholedochal approach. Postoperatively, the hospital stay, wound infection, LFTs abnormalities or pancreatitis were all looked for as well as bile leak, cholangitis, bleeding and follow up data for stone recurrence.

### Operative technique

All patients received prophylactic antibiotic and DVT prophylaxis according to the local policy. The procedure was done in supine position with adjustable operative bed to allow fluoroscopic C- arm for imaging the patient’s right upper quadrant. The procedure was carried out using a 4-trocars laparoscopic cholecystectomy technique. After dissecting the Calot’s triangle, the cystic artery was clipped and transected. A clip was applied on the gall bladder side of the cystic duct to prevent stone slippage either into CBD or the operative field while the cranial side was left connected with the CBD. A small diameter catheter was introduced through a small nick in the cystic duct down to the CBD to flush it and to do intraoperative cholangiogram to look for filling defect, its size, site, and number and free passage of contrast into the duodenum or any anatomical variation if any.

### Transcystic clearance (103 patients)

**Fig. 1:**
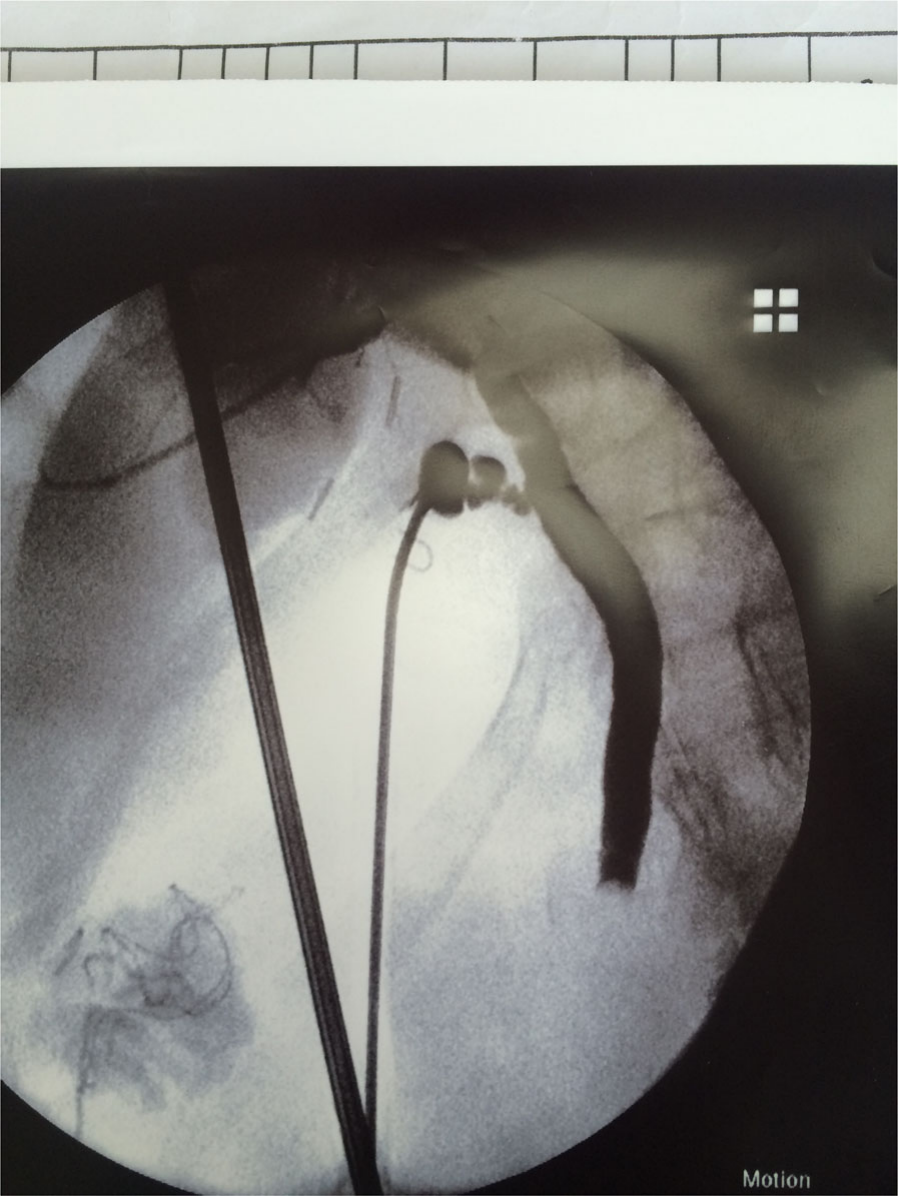
intraoperative cholangiogram showing a filling defect that prevents passage of contrast into the duodenum

**Fig. 2:**
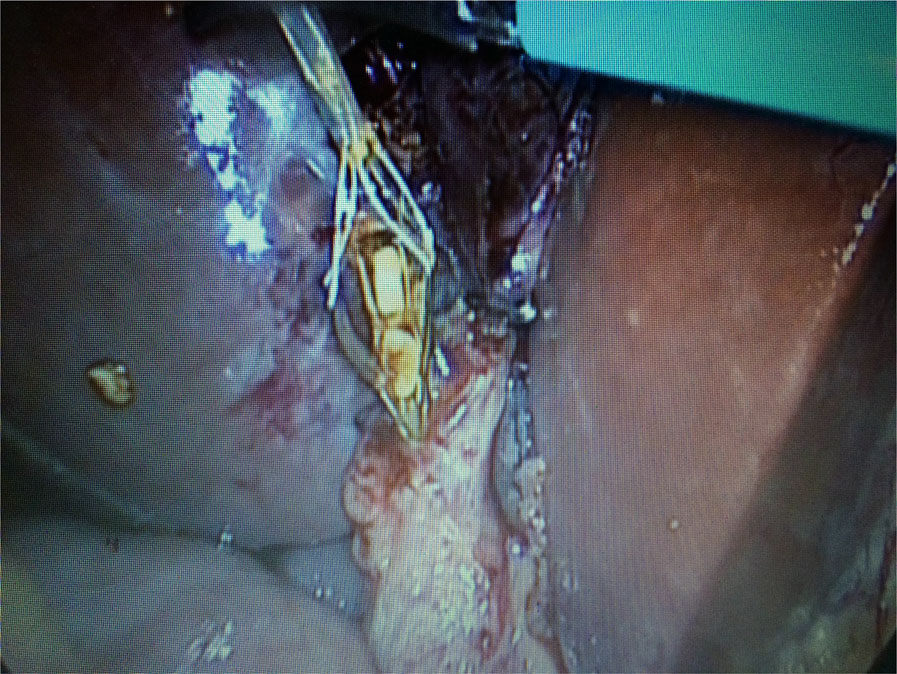
Trans-cystic stone extraction by Dormia basket

After dilating the cystic duct and on a guide wire, the CBD stones were extracted by flushing or with help of Dormia basket or balloon catheter to pull stones into the intra-abdominal cavity to be retrieved. A complete stone clearance was confirmed by completion cholangiogram or choledechoscope. The CBD side of the cystic duct was clipped and the gall bladder was removed in the usual manner (Figs. 1 and 2).

### Choledochotomy approach (26 patients)

**Fig. 3:**
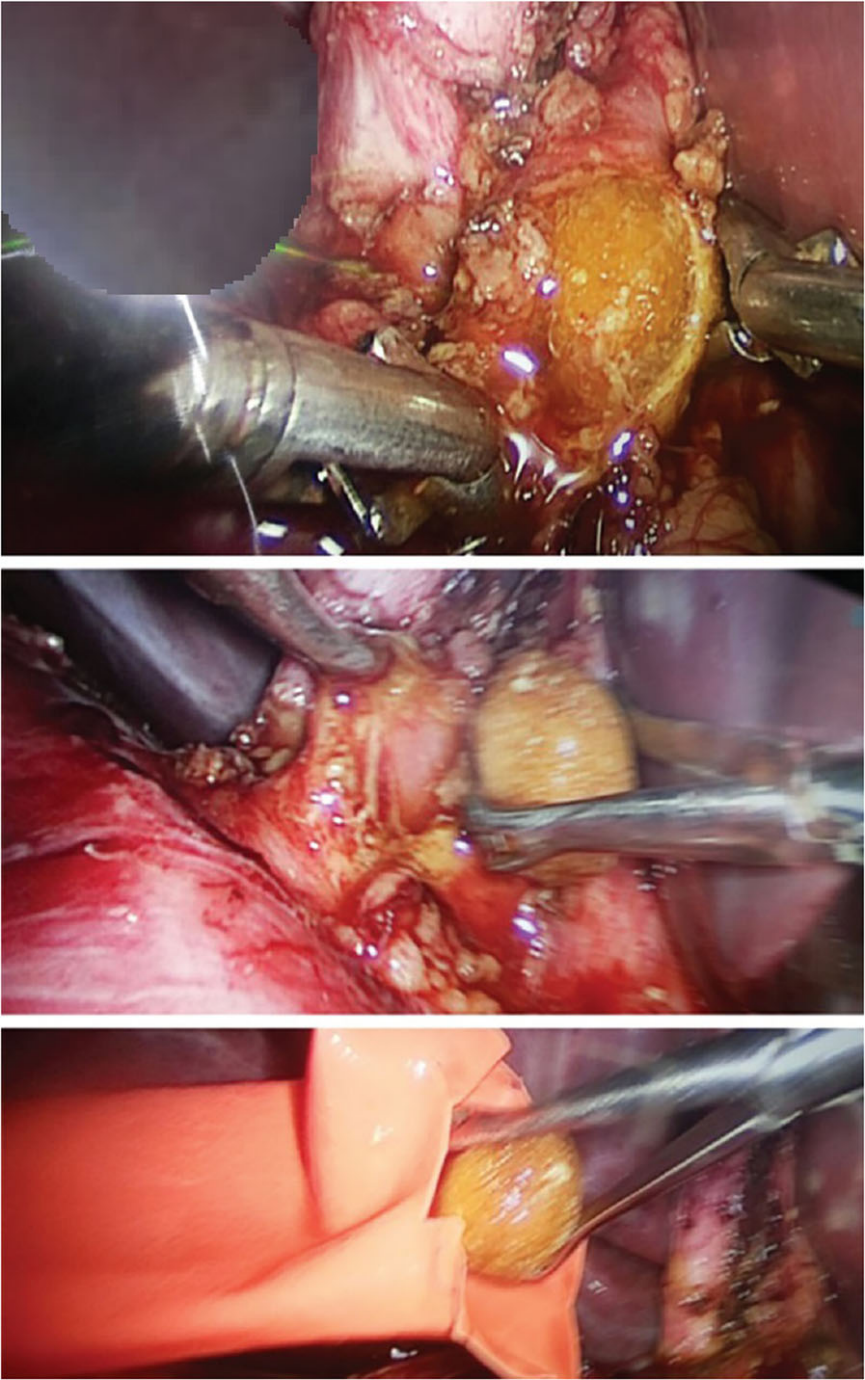
Choledocotomy technique and extraction of big CBD stone

**Fig. 4:**
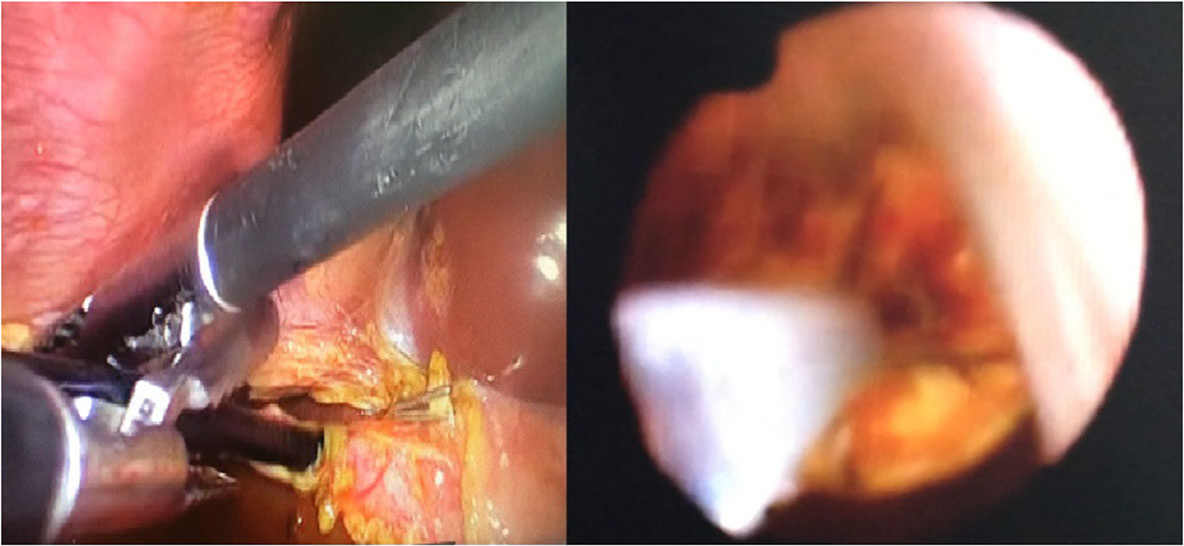
Intraoperative trans-cystic Choledocoscope

This approach was used either after failed trial of cannulation of cystic duct or failed stone extraction through cystic duct, in three patients, we employed it without trying the trans-cystic technique because they were post failed ERCP due to big impacted stones. IOC was done through CBD puncture with lumber needle which showed CBD stones. In these cases we converted to choledochotomy in which the CBD was exposed and a vertical ductotomy was done on the anterior surface of the duct below the junction between cystic duct and CBD (Fig. 3). The techniques for stone clearance are identical to the trans-cystic approach. Through the choledochoscope, flushing with saline was done under pressure to facilitate clearance of small stones. Dormia basket and/or balloon catheter can be also used to pull stones to the abdominal cavity then to be retrieved outside (Fig. 4). The choledochotomy was closed over a T-tube in nine cases and over an antegrade stent in 10 cases; the stent was inserted through the site of cholodotomy under guidance of fluoroscopy for removal by ERCP later. Primary closure of the CBD was done in the remaining seven patients with absorbable suture. External tube drains were used only when we perform choledochotomy technique and not used in trans-cystic technique.

### Statistics

All data were prospectively collected in designed sheets and were reviewed retrospectively for statistical purposes. Continuous variables were compared using a Student *t*-test or Mann-Whitney test, as appropriate. Chi-square test was used for comparing categorical variables. *P* < 0.05 was considered statistically significant. Data are expressed as mean (SD). Statistical analysis was performed using a commercially available software package (SPSS version 11.5 for Windows; SPSS Inc, Chicago, IL).

## Results

From April 2011 to June 2016, we performed 3012 laparoscopic cholecystectomy at our institutes. 295 (9.8%) out 3012patients have undergone intraoperative cholangiogram and 129 (4.3%) were diagnosed to have choledocholithiasis. Male to female ratio was 43/86, with mean age of 37 ± 8 years (range 27 to 57 years) (Table 1). Transcystic exploration was done in 103 patients. Transcholedochal route was performed in 26 patients, 13 patients had too big CBD stones to be extracted through cystic duct, and 11 had failed cannulation of the cystic duct while in three patients choledochotomy was employed from the start due to post failed ERCP clearance of big impacted stones. In transcholedochal LCBDE group (26 patients), primary closure of CBD was done in seven patients, nine patients had CBD closed over T-tube whereas 10 patients closed over stent. All patients survived the operation with 0% conversion rate to open surgery. Six patients underwent postoperative ERCP because of incomplete CBD clearance (4 cases), symptomatic stenosed papilla (2 cases). The duration of the operation was 126 min (range, 102–140 min) in both trans-cystic and choledochotomy techniques. Intraoperative blood loss was minimal (less than 10 ml) with no intraoperative complications (Table 2).

**Table 1:** Patients’ characteristics and preoperative parameters

Variable	Patients *N* = 129
Age	37 ± 8 years (range 27 to 57 years)
Gender (female/male)	93/36
Weight	69 ± 13 (59–126 kg)
Height	157 ± 15 (149–178 cm)
BMI	29 ± 6 (23–46)
ASA	
ASA I	76 (59%)
ASA II	47 (36.4%)
ASA III	6 (4.6%)
Hx of biliary pain	59 (45.7%)
Hx of jaundice	9 (7%)
Hx of pancreatitis	13 (10%)
Mean bilirubin	1.003 ± 0.003 (0.3–3.2 mg)
Mean ALP	175 ± 19 (71–396 U)
Mean ALT	209 ± 32 (31–702 U)
Mean ALT	195 ± 29 (29–560 U)
Mean CBD diameter	7.3 ± 1.7 (4–19 mm)

*ALP* alkaline phosphatase, *ALT* Alanine transaminase, *AST* Aspartate transaminase, *hx* history, *ASA* American Society of Anesthesiologists

**Table 2:** Operative outcomes

Variable	Patients *N* = 129
Lap. Cholecystectomy	3012
IOC	295 (9.8%)
LCBDE	129 (4.3%)
Trans-cystic	103 (78.8%)
Trans-choledochotomy	26 (21.2%)
T-tube	10
Stent	9
Primary closure	7
Operative time	126 (range, 102–140 min)
LC	39 ± 13 (25–59 min)
Trans-cystic	40 ± 16 (37–63 min)
Trans-choledochotomy	59 ± 19 (47–109 min)
Mean No. of CBD stones	2.4 ± 1.6 (1–7)
Mean size of stones	4.2 ± 1.3 (3–15 mm)
Mean blood loss	10 ± 3.6 (5–50 ml)
Blood transfusion	0

*IOC* intraoperative cholangiogram, *CBD* common bile duct, *LCBDE* laparoscopic CBD exploration, *LC* laparoscopic cholecystectomy

During patients’ stay, patients’ assessment was done clinically and by laboratory evaluation. Transient colic pain occurred in two patients with elevated liver enzymes. One was managed conservatively and the other patient had sustained severe biliary colic for 2 days after her operation and sent for higher hospital to do ERCP which revealed very tight papilla without any CBD stones, sphincterotomy cured the problem. A transient increase in the LFTs (AST & ALT) was observed in three patients, it went back to normal on postoperative day 3 with no treatment (Table 3). All patients had an uneventful postoperative period and were discharged home with median hospital stay of 4 (range;1–15 days). In trans-cystic approach group, the patients were discharged home on day 3–4 postoperatively once we were completely assured that the operation. If T-tube was inserted, a post-operative cholangiogram was done after 7–10 days and if free, the tube was clamped and left in place for total 14 days. No haemobilia, abdominal bleeding, or pancreatitis occurred in our series.

**Table 3:** Postoperative outcomes

Variable	Patients *N* = 129
Return to oral intake	6 ± 2.3 (4–19 h)
Return to activity	17 ± 5.3 (6–29 h)
Hospital stay	4 (range;1–15 days)
-Trans-cystic	2 (range;1–3 days)
-Trans-choledochectomy	6 (range; 4–15 days)
Mean postop. bilirubin	0.6 ± 0.03 (0.4–3.2 mg)
Mean post-op. ALP	129 ± 18 (74–293 U)
Mean post-op. ALT	113 ± 11 (23–174 U)
Mean post-op. AST	159 ± 19 (19–163 U)
Mean T-tube cholangiogram	5.2 ± 3 (4–8 days)
T-tube removal	15.7 ± 4 (13–25 days)
Complications (I,II)	
-Pancreatitis (II)	0
-Cholangitis (II)	0
-Hemobilia (II)	0
-Accidental T-tube displacement (II)	0
-Transient liver enzymes elevation (I)	3 (2.3%)
-Wound infection (II)	3 (2.3%)
-Choleperitoneum (II)	1 (0.76%)
-Pneumonia (II)	0
-Biliary colic (II)	2 (1.5%)
-Abdominal bleeding (II)	0

*ALP* alkaline phosphatase, *ALT* Alanine transaminase, *AST* Aspartate transaminase, ^*^ Dindo-Clavien classification

Upon discharge, patients were instructed for first follow up after 1 week to have stitches and T-tube removed provided cholangiogram was unremarkable. Schedule of follow up was then given for patients every 6 months during the first year and annually thereafter. Clinical evaluations and liver function tests were done every visit and additional imaging studies were requested if needed. In one patient, T-tube was removed on day 11 post-operatively, but she developed bile leak after removal which necessitated 2 extra days admission, had been treated conservatively and discharged home in good condition. Another patient, female 37 years old, in choledochotomy group with T-tube closure, the catheter 6 Fr failed to pass through the papilla. The average T-tube drainage was around 400 ml bile and her postoperative cholangiogram showed tight papilla with no residual stones, she had been sent to another hospital to have ERCP which revealed no residual stones and sphincterotomy was sufficient. Her T-tube was clamped for one more week and removed after normal cholangiogram. Three patients developed wound infection (umbilical port) that was treated conservatively. There were no mortalities and all procedures had been completed laparoscopically with no conversion to open.

The mean follow up was 9 ± 3.4 months (2–39 months). No patients showed retained CBD stones by clinical, laboratory and imaging studies. We used the Dindo-Clavien classification to stratify the severity of operative and postoperative complications [7].

## Discussion

If 9–16% of patients who have cholelithiasis harbour concomitant CBD choledocholithiasis, so it is a considerable problem that requires a proper strategy to solve. Options are many, starting from open cholecystectomy with open CBD exploration to laparoscopic cholecystectomy with pre- or post-operative ERCP then laparoscopic cholecystectomy with LCBDE. The introduction of laparoscopic CBD exploration has made it possible to avoid the drawbacks of both two-stages procedure (laparoscopic cholecystectomy plus pre- or post-operative ERCP) and also the drawbacks of the open CBD exploration [8, 9]. LCBD were not commonly used by surgeons till very late after introduction of laparoscopic cholecystectomy. The reasons for this delay are because it needs instrumental difficulties as well as the rely of most of surgeons on the alternative interventional methods especially ERCP because LCBDE is technically demanding and needs high level of experience [10, 11].

In this study, we present our preliminary experience of LCBDE in our institutes with success rate of 95.4% (123/129) which are comparable to the results of ERCP & open CBD exploration with less morbidity and mortality and hopefully the success rate will increase with increasing experience [12, 13].

The trans-cystic approach is technically easier, feasible, and less invasive with better patients’ satisfaction. Surgeons usually try it first but it has its limitations and indications e.g. dilated cystic duct, small stones (preferably single stone) and there should be no stent in the CBD [3, 11–13]. We performed this approach in 103/129 (79.8%) patientswith success rate of 98/103 (95%) which is almost comparable to others. Whenever this approach is difficult or impossible, we converted to trans-choledochal one. The choledochotomy approach is technically demanding and needs advanced laparoscopic experience [6, 14, 15]. 29/129 (20.2%) patients have been undergone LCBDE through transcholedochal route with 28/29 (96.6%) success rate. We performed this choledochotomy approach after failed trials of trans-cystic route either due to failed cannulation or failed stone extraction through cystic duct. Choledochotomy technique was our first choice without trying the trans-cystic technique in three patients with failed ERCP due to impacted big stone in the CBD.

We routinely used intraoperative cholangiogram before stone extraction for confirmation the presence of stones and after to make sure of complete clearance of the CBD. Choledocoscope is a very helpful tool in CBD exploration, in both direct visualization of the intraluminal stones and also in removing them by the aid of Dormia basket or Fogarty’s vascular catheter [8, 16–18]. We used the choledocoscope in most of our cases to confirm the complete clearance of the CBD and to inject saline for washout of stone fragments and debris.

In our study we closed the choledocotomy over T-tube in 10/26 cases (38.5%). Closure over T-tube provides biliary decompression especially when there was concern about retained fragments or tiny stones, it enables us for imaging the biliary system postoperatively and it provides an access through which any retained stones can be removed. T-tube has its disadvantages, it might make a way for bacteraemia, accidental premature dislodgment, obstruction and it might be associated with bile leak and peritonitis at its removal. Closure over stent was used in 9/26 cases (34.6%) which provides safe closure of choledochotomy as it provides biliary decompression same as T-tube does without its disadvantages, however, it needs follow up ERCP to remove it later. Finally, primary closure without stent was used in 7/26 cases (26.9%) and wehave no bile leakage in our cases and we have no intra-abdominal collections as well.

However, the length of stay for the laparoscopic cholecystectomy is generally short (from 1 to 3 days), it is longer for laparoscopic CBD exploration 1–7 days in most of studies [12, 18–20]. In our study, the length of stay depended upon the technique employed; it was 1–3 days in trans-cystic group and longer (4–15 days) in choledocotomy one especially when we used T-tube to close the choledocotomy.

In most of studies the mortality of laparoscopic CBD exploration is 0 to 1% in the hands of experienced biliary surgeons. This rate is similar to the incidence found in open CBD exploration [21–25]. In our study, we have no reported mortality cases, which may be attributed to improved preoperative preparation, improved anaesthesia and selection of cases.

Our study has some limitations, it is a retrospective study and showed a wide heterogeneity among the surgeons’ experience in performing this LCBDE. The sample size of patients enrolled in this study is relatively small. This study represents our initial experience in preforming LCBDE, this represents a limitation per say. We recommend for another study to be done in a prospective way and to enrol a much larger sample size.

Laparoendoscopic rendezvous is a promising alternative and has many pros; it provides selective cannulation of CBD and avoids both inadvertent cannulation of pancreatic duct with subsequent accidental injection of the contrast under pressure into the pancreatic duct [26]. We had six patients underwent postoperative ERCP, 4 because of incomplete CBD and two cases due to symptomatic stenosis of the papilla. Some hospitals at which this study was conducted are lacking for ERCP services. ERCP is crucial because it is the cornerstone for LCBDE, it provides an alternative optionfor patients with CBD stones synchronous with gall bladder stones and relieved stenosis when present as shown in our study. Therefore a limitation in our study is the lack of access of ERCP for the six patients due to unavailability in hospitals (two patients) and gastroenterologists (four patients). However, if available, Laparoendoscopic rendezvous would have been an excellent intervention for these six patients.

## Conclusion

Laparoscopic CBD exploration is an effective single stage procedure for the treatment of GB and CBD stone in one session making use of the benefits of minimally invasive approach and avoiding the drawbacks of ERCP as well as open CBD approach. LCBDE can be performed after proper training and adequate equipment and laparoscopic facilities.

## Abbreviations

ASA: American Society of Anesthesiologists
BMI: Body mass index
CBD: Common bile duct
DVT: Deep vein thrombosis
ERCP: Endoscopic retrograde cholangiopancreatography
GB: Gall bladder
IOC: Intraoperative cholangiogram
LC: Laparoscopic cholecystectomy
LCBDE: Laparoscopic common bile duct exploration
LFTs: Liver function tests
MRCP: Magnetic resonance cholangiopancreatectomy
